# A Compact Thévenin Model for a Rectenna and Its Application to an RF Harvester with MPPT

**DOI:** 10.3390/s19071641

**Published:** 2019-04-06

**Authors:** Manel Gasulla, Edgar Ripoll-Vercellone, Ferran Reverter

**Affiliations:** 1 e-CAT Research Group, Department of Electronic Engineering, Castelldefels School of Telecommunications and Aerospace Engineering, Universitat Politècnica de Catalunya, c/ Esteve Terradas, 7, 08860 Castelldefels (Barcelona), Spain; edgar.ripoll.vercellone@upc.edu (E.R.-V.); ferran.reverter@upc.edu (F.R.); 2 Idneo Technologies, c/ Rec de Dalt s/n., 08100 Mollet del Vallès (Barcelona), Spain

**Keywords:** RF harvesting, rectenna, Thévenin model, maximum power point tracking, MPPT, L-matching network, sensor node

## Abstract

This paper proposes a compact Thévenin model for a rectenna. This model is then applied to design a high-efficiency radio frequency harvester with a maximum power point tracker (MPPT). The rectenna under study consists of an L-matching network and a half-wave rectifier. The derived model is simpler and more compact than those suggested so far in the literature and includes explicit expressions of the Thévenin voltage (*V*_oc_) and resistance and of the power efficiency related with the parameters of the rectenna. The rectenna was implemented and characterized from −30 to −10 dBm at 808 MHz. Experimental results agree with the proposed model, showing a linear current–voltage relationship as well as a maximum efficiency at *V*_oc_/2, in particular 60% at −10 dBm, which is a remarkable value. An MPPT was also used at the rectenna output in order to automatically work at the maximum efficiency point, with an overall efficiency near 50% at −10 dBm. Further tests were performed using a nearby transmitting antenna for powering a sensor node with a power consumption of 4.2 µW.

## 1. Introduction

Radio frequency (RF) energy harvesting has been extensively proposed to power tiny devices such as RFID tags, autonomous sensors, or Internet of Things (IoT) nodes. RF energy can be harvested either from dedicated sources, such as in the case of RFID devices [1,2,3,4], or from the RF energy already present in the ambient environment and coming from unintentional sources such as TV, FM radio, cellular, or WiFi emitters [2,5,6,7,8,9,10].

**Figure 1:**
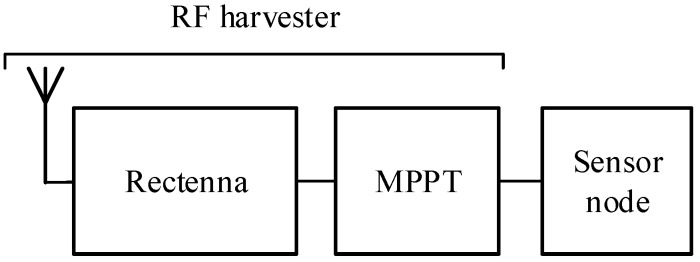
Block diagram of a radio frequency (RF) harvester powering a sensor node.

Figure 1 shows the block diagram of an RF harvester powering a sensor node. The rectenna (rectifying antenna) transforms the RF signal to a DC voltage and the maximum power point tracker (MPPT) provides the optimum load to the rectenna to transfer the maximum power to the sensor node.

The rectenna is composed of an antenna, an impedance matching network, and a rectifier. As the available power at the antenna decreases so does the generated voltage. Whenever this voltage is not high enough to properly bias the diodes of the rectifier, power efficiency severely decreases. Several techniques have been proposed to increase the efficiency at low power levels. One of them consists of using an L-matching network for boosting the voltage at the rectifier input [1,3,5,10,11,12,13,14,15,16,17,18,19,20,21]. As for the MPPT, several works propose its use with rectennas using either commercial chips [6,7] or ad hoc designs [22,23,24,25].

With the aim of gaining more insight into the performance of the rectennas, different analytical models are proposed. However, the derived expressions, which in some cases seek to model the rectenna output as an equivalent Thévenin circuit, are rather complex and may require additional simulations or extensive calculations, which hide the influence of the different parameters of the rectenna on its performance [12,18,26,27,28]. At the other extreme, the Thévenin parameters are sometimes inferred by experimental characterization [25,29,30,31,32]. However, in these cases no relationship with the rectenna parameters is established.

Taking into account the previous limitations, this paper proposes a compact Thévenin model for the rectenna with the benefit of achieving manageable expressions of the Thévenin parameters as a function of the parameters of the rectenna so as to gain insight into its operation. In particular, the rectenna under study consists of an L-matching network and of a half-wave rectifier. The proposed model is then experimentally verified and the rectenna further tested in a high-efficency RF harvester with MPPT.

The paper, which continues and expands the work presented in [32], is organized as follows. Section 2 presents the rectenna and the derived Thévenin equivalent. Section 3 describes the MPPT and the sensor node. Section 4 presents the materials and methods and Section 5 provides the experimental results and discussions. Finally, Section 6 concludes the work. Complementarily, two appendices are included. Appendix A presents an analytical development useful for the derivation of the Thévenin equivalent and Appendix B shows simulations of the rectenna with and without the matching network.

## 2. Rectenna and Its Thévenin Model

**Figure 2:**
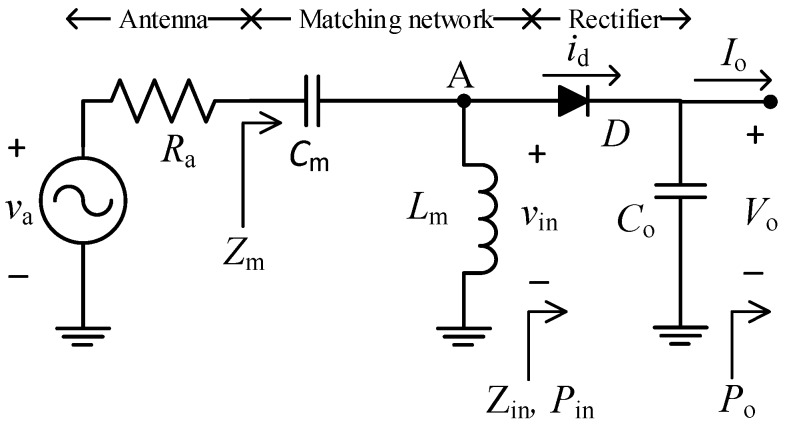
Schematic circuit of the rectenna under study.

Figure 2 shows the schematic circuit of the rectenna under study [33], which includes a high-pass L-matching network (composed of a capacitor *C*_m_ and an inductor *L*_m_), a half-wave rectifier, and an output filtering capacitor (*C*_o_). The antenna is modelled by a sinusoidal voltage source *v*_a_ of amplitude *V*_ap_ and frequency *f*_o_ with a series radiation resistance *R*_a_. On the other hand, *v*_in_, *Z*_in,_ and *P*_in_ are, respectively, the sinusoidal voltage, impedance, and power at the input of the rectifier, *i*_d_ is the diode current, and *V*_o_, *I*_o_, and *P*_o_ are, respectively, the DC voltage, current, and power at the rectenna output. An equivalent resistance *R*_o_ is defined as *V*_o_/*I*_o_.

The amplitude *V*_ap_ is given by [12] as follows:

(1) $$V_{\mathrm{ap}}=2\sqrt{2R_{a}P_{\mathrm{av}}},$$

where *P*_av_ is the available power at the antenna. The matching network, at matching conditions, that is, *Z*_m_ = *R*_a_ (where *Z*_m_ is defined in Figure 2), boosts the voltage at the input of the rectifier by a voltage gain, *G*_t_, given by [33] as follows:

(2) $$G_{t}=\frac{V_{\mathrm{inp}}}{V_{\mathrm{ap}}}=\frac{1}{2}\sqrt{\left(1+Q^{2}\right)},$$

where *V*_inp_ is the voltage amplitude of *v*_in_ and *Q* is the circuit quality factor given by:

(3) $$Q=\frac{1}{\omega_{o}C_{m}R_{a}},$$

where *ω*_o_ = 2π*f*_o_. On the other hand, the value of *L*_m_ must comply:

(4) $$L_{m}=\frac{1}{\omega_{o}^{2}}\frac{1}{C_{p}+C_{m}Q^{2}/\left(1+Q^{2}\right)},$$

where *C*_p_ models the parasitic capacitance between node A and ground.

**Figure 3:**
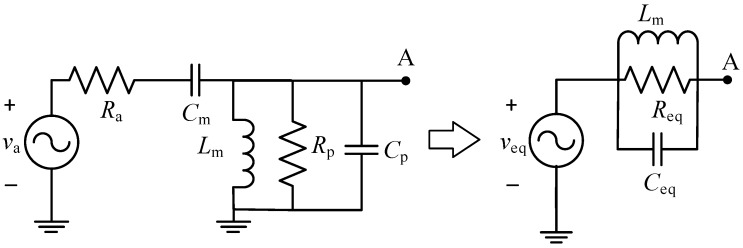
(**left**) Equivalent input circuit of the antenna and L-matching network considering the parasitic effects of the coil and diode and (**right**) its Thévenin equivalent circuit.

To ease the analysis of the proposed rectenna and also gain more insight into its performance, a compact Thévenin model is provided here. First, the left-hand equivalent circuit of Figure 3 accounts for the antenna, the matching network, and the parasitic elements (*R*_p_-*C*_p_) of the coil, diode, and layout of the circuit. These parasitic elements are derived in Appendix A, where *R*_p_ models the losses of the coil and diode and *C*_p_ includes the parasitic capacitance of the diode, coil, and layout.

Analyzing the left-hand circuit of Figure 3 at *f*_o_, we can achieve the Thévenin equivalent represented by the right-hand circuit of Figure 3, where:

(5) $$\begin{array}{c} v_{\mathrm{eq}}=2G_{t}v_{a}\frac{R_{p}}{4G_{t}^{2}R_{a}+R_{p}},\\ R_{\mathrm{eq}}=\left(4G_{t}^{2}R_{a}\right)\|R_{p},\\ C_{\mathrm{eq}}=C_{m}\frac{Q^{2}}{1+Q^{2}}+C_{p}. \end{array}$$

**Figure 4:**
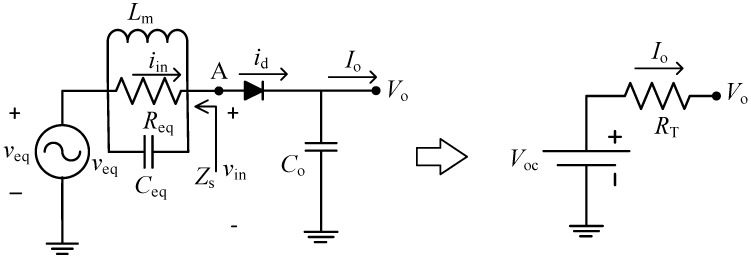
(**left**) Equivalent circuit of the rectenna using the right-hand circuit of Figure 3 and (**right**) its Thévenin equivalent.

Next, the Thévenin equivalent of Figure 3 is linked to the next stage of the rectenna, the rectifier, resulting in the left-hand circuit of Figure 4, where the diode does not include its parasitic elements since they have been already considered in the previous derivation (they are included in *R*_eq_ and *C*_eq_). The diode is forward biased when *v*_in_, assumed sinusoidal, surpasses *V*_o_. As a result, *i*_d_ is pulsed and is composed of the fundamental frequency (*f*_o_) as well as its harmonics and a DC component (*I*_o_). Impedance *Z*_s_ (defined in the circuit) is zero at DC (due to the coil *L*_m_) and is equal to *R*_eq_ at *f*_o_ since *L*_m_ and *C*_eq_ form a parallel resonant circuit presenting an infinite impedance. On the other hand, at the harmonics of *f*_o_ we have *Z*_s_ << *R*_eq_ (due to *C*_eq_) whenever *Q* is high enough. Therefore, only the current at *f*_o_ (*i*_in_) originates a voltage drop and *v*_in_ will be sinusoidal, as assumed before. Thus, apart from boosting the voltage, the matching network ideally acts as an input band-pass filter that prevents any of the DC current and harmonics to flow through the antenna resistance and dissipate power. This leads to an ideal rectenna efficiency of 100%, assuming no losses in the circuit components and in the diode [34]. Contrariwise, when no matching network is present, maximum rectenna efficiency decreases to 46%, due to the additional losses at *R*_a_ originated by the current harmonics generated by the diode pulsed current, as demonstrated in [35]. Appendix B confirms these results via simulations. Finally, the value of *C*_o_ has to be much higher than the diode junction capacitance (*C*_j_), as explained in Appendix A, to keep *V*_o_ nearly constant, that is, with a low voltage ripple (Δ*V*_o_). This second condition leads to:

(6) $$C_{o}>\frac{I_{o}}{\Delta V_{o}f_{o}}.$$

The left-hand circuit of Figure 4 leads to the equivalent Thévenin circuit of the rectenna, represented by the right-hand circuit of Figure 4, by linking their output voltage–current relationship. For the left-hand circuit, we have:

(7) $$V_{o}=V_{\mathrm{inp}}-V_{\gamma },$$

assuming a fixed forward voltage drop *V*_γ_ at the diode and:

(8) $$V_{\mathrm{inp}}=V_{\mathrm{eqp}}-R_{\mathrm{eq}}I_{\mathrm{inp}},$$

where *V*_eqp_ and *I*_inp_ are the amplitudes of *v*_eq_ and *i*_in_, respectively. Substituting (8) into (7) provides:

(9) $$V_{o}=V_{\mathrm{eqp}}-V_{\gamma }-R_{\mathrm{eq}}I_{\mathrm{inp}}.$$

On the other hand, for the right-hand circuit we have:

(10) $$V_{o}=V_{\mathrm{oc}}-R_{T}I_{o}.$$

Then, by equating powers, we obtain:

(11) $$P_{\mathrm{in}}=P_{o}+P_{d},$$

where

(12) $$\begin{array}{c} P_{\mathrm{in}}=\frac{V_{\mathrm{inp}}I_{\mathrm{inp}}}{2},\\ P_{o}=V_{o}I_{o},\\ P_{d}=V_{\gamma }I_{o}, \end{array}$$

and *P*_d_ is the average power dissipated across the diode. Thus, replacing (12) into (11) and using (7), we arrive at the following:

(13) $$I_{\mathrm{inp}}=2I_{o}.$$

Finally, using (13) in (9) and equating (9) and (10), we obtain the parameters of the Thévenin model:

(14) $$\begin{array}{c} V_{\mathrm{oc}}=V_{\mathrm{eqp}}-V_{\gamma },\\ R_{T}=2R_{\mathrm{eq}}, \end{array}$$

where *V*_eqp_ can be derived from *v*_eq_ in (5), using *V*_ap_ instead of *v*_a_, resulting in:

(15) $$V_{\mathrm{eqp}}=2G_{t}V_{\mathrm{ap}}\frac{R_{p}}{4G_{t}^{2}R_{a}+R_{p}}.$$

Then, using (15) and *R*_eq_ of (5) in (14), we have:

(16) $$\begin{array}{c} V_{\mathrm{oc}}=2G_{t}V_{\mathrm{ap}}\frac{R_{p}}{4G_{t}^{2}R_{a}+R_{p}}-V_{\gamma },\\ R_{T}=2\left[\left(4G_{t}^{2}R_{a}\right)\|R_{p}\right]. \end{array}$$

Therefore, from (16), with an increasing *P*_av_ and thus *V*_ap_, *V*_oc_ increases whereas *R*_T_ holds constant. Next, from (10), we can express *I*_o_ as:

(17) $$I_{o}=\left(V_{\mathrm{oc}}-V_{o}\right)/R_{T},$$

and the output power *P*_o_ over a load resistor *R*_o_ can be simply calculated as:

(18) $$P_{o}=V_{o}I_{o}=\frac{V_{\mathrm{oc}}V_{o}-V_{o}^{2}}{R_{T}},$$

being the power efficiency of the rectenna as:

(19) $$\eta_{\mathrm{rect}}=\frac{P_{o}}{P_{\mathrm{av}}}=\frac{V_{\mathrm{oc}}V_{o}-V_{o}^{2}}{P_{\mathrm{av}}R_{T}}.$$

Applying the maximum power transfer theorem, maximum power is extracted from the rectenna for *V*_o_ = 0.5*V*_oc_, which is known as the maximum power point (MPP) voltage (*V*_MPP_). From (19), the resulting efficiency is as:

(20) $$\eta_{\mathrm{rect},\max}=\frac{V_{\mathrm{oc}}^{2}}{4P_{\mathrm{av}}R_{T}}.$$

Thus, using (16) in (20), we arrive at:

(21) $$\eta_{\mathrm{rect},\max}=\frac{R_{p}}{4G_{t}^{2}R_{a}+R_{p}}{\left(1-\frac{V_{\gamma }}{\sqrt{2R_{a}P_{\mathrm{av}}}}\frac{4G_{t}^{2}R_{a}+R_{p}}{4G_{t}R_{p}}\right)}^{2}.$$

As can be seen from (21), *η*_rect,max_ increases with increasing *P*_av_. Obviously, with no losses (*R*_p_ = ∞ and *V*_γ_ = 0) *η*_rect,max_ = 1 is obtained. On the other hand, the dependence of *η*_rect,max_ on *G*_t_ is rather more complex. In [33], an optimum value of *G*_t_ was derived arising from the trade-off between the losses introduced by the coil and that due to the voltage drop of the diode. This optimum gain leads, from (16), to a particular value of *R*_T_.

## 3. MPPT and Sensor Node

In general, a sensor node directly connected to the output of the rectenna will not provide an equivalent resistance *R*_o_ = *R*_T_, at which the rectenna output operates at the MPP. Thus, an impedance matching stage (in addition to the matching network of the rectenna) is needed between the rectenna output and the sensor node, which can be implemented by a DC/DC converter. An MPPT, which consists of a DC/DC converter plus a tracking algorithm, can be used for automatically searching and settling that optimum value of *R*_o_, which also corresponds to *V*_o_ = *V*_MPP_. Thus, the overall power efficiency of the RF harvester will be given by the following:

(22) $$\eta_{T}=\eta_{\mathrm{rect},\max}\eta_{\mathrm{MPPT}},$$

where *η*_MPPT_ is the efficiency of the MPPT and *η*_rect_ = *η*_rect,max_ since the MPPT biases the rectenna at the MPP.

In this work, the fractional open circuit voltage (FOCV) MPPT technique is used, since it leads to simple and power efficient implementations. In this technique, the open circuit voltage (*V*_oc_) of the energy transducer (a rectenna here) is first measured and a fraction *k* of *V*_oc_ is used to operate at *V*_MPP_ and thus achieve *η*_rect,max_. Taking into account the analysis in Section 2, a proper choice here is *k* = 0.5 (*V*_o_ = *V*_MPP_ = 0.5*V*_oc_).

**Figure 5:**
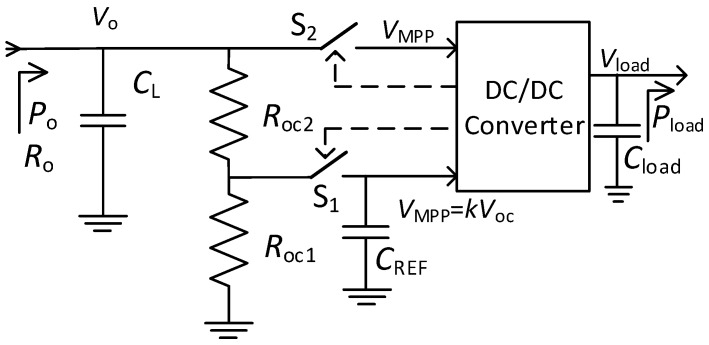
Block diagram for the implementation of the fractional open circuit voltage maximum power point tracking technique.

Figure 5 presents the block diagram for the implementation of the FOCV MPPT technique, where *C*_L_, *C*_REF_, and *C*_load_ are capacitors, *R*_oc1_ and *R*_oc2_ are resistors, S_1_ and S_2_ are switches, *V*_load_ is the output voltage used to power the sensor node, and *P*_load_ is the power transferred to the sensor node. The operation is the following. First, S_1_ closes and S_2_ opens (sampling period). For high values of *R*_oc1_ and *R*_oc2_, the output of the rectenna can be considered as open and thus *V*_o_ = *V*_oc_. The voltage divider formed by *R*_oc1_ and *R*_oc2_ fixes *V*_MPP_ = *kV*_oc_, being *k* = 0.5 here (i.e., *R*_oc1_ = *R*_oc2_). The input capacitor (*C*_L_) momentarily stores the incoming harvested energy. Secondly, S_1_ opens and S_2_ closes (regulation period). Thus, V_MPP_ holds constant thanks to *C*_REF_, and the DC/DC converter settles *V*_o_ around *V*_MPP_ and transfers the harvested energy by the rectenna to the sensor node.

In order to periodically update *V*_oc_ (i.e., a change in *P*_av_ changes *V*_oc_), the described sequence is periodically repeated, with the sampling period being much shorter than the regulation period. In this way, *V*_o_ will settle most of time at *V*_MPP_. To increase the efficiency at light loads, the DC/DC converter uses special control techniques such as pulse frequency modulation (PFM) or burst-mode [36].

Taking into account (22), *P*_load_ can be related with *P*_av_ as follows:

(23) $$P_{\mathrm{load}}=\eta_{T}P_{\mathrm{av}}.$$

The value of *P*_load_ and thus of *P*_av_ must be enough, in average, to power the sensor node, which usually includes a rechargeable storage unit. This unit accounts for the variability of *P*_av_, gathering or providing energy whenever *P*_av_ is higher or lower than required. Storage units can be supercapacitors, batteries, or a combination of both [37]. On the other hand, the required value of *P*_load_ and thus of *P*_av_ can be reduced by operating the sensor node in sleep mode most of the time and minimizing its active time.

## 4. Materials and Methods

The rectenna shown in Figure 2 was implemented on a printed circuit board with Rogers substrate and with the following components: *C*_m_ = 0.5 pF (AVX, Fountain Inn, SC, USA), *L*_m_ = 27 nH (0603CS model, Coilcraft, Cary, IL, USA), *C*_o_ = 1 nF, and a Schottky HSMS-2850 diode (Avago Technologies, San Jose, CA, USA) [33]. The selected value of *C*_o_ comfortably accomplished, in order to theoretically have a small ripple (below 1 mV) with the values of *I*_o_ shown later in Section 5, as well as the condition stated in Appendix A (*C*_o_ >> *C*_j_). The circuit of Figure 2 was used for the rectenna characterization, where an RF generator (Agilent E4433B, Santa Clara, CA, USA) was connected at the input instead of the antenna and a Source Measure Unit (SMU, Agilent B2901A, Santa Clara, CA, USA) configured as a voltage sink (quadrant IV) at the output. The generator was set at a tuned optimal frequency of 808 MHz and at different values of *P*_av_ (−30 dBm, −20 dBm, and −10 dBm). For each value of *P*_av_, the SMU was set at different values of *V*_o_ while measuring *P*_o_. Then, *η*_rect_ was obtained as *P*_o_/*P*_av_.

As for the FOCV MPPT, a BQ25504 chip (Texas Instruments, Dallas, TX, USA) was used, and in particular an evaluation board provided by the manufacturer. The chip contains a boost converter with PFM control and the board includes, in reference to Figure 5, *C*_L_ = 4.8 µF (combination of two ceramic capacitors of 4.7 μF and 100 nF placed in parallel), *C*_REF_ = 10 nF, and *C*_load_ = 104.8 µF (combination of three ceramic capacitors of 100 μF, 4.7 μF, and 100 nF placed in parallel). The default values of *R*_oc1_ and *R*_oc2_ were modified to 10 MΩ in order to fix *k* = 0.5 (the default value is set to 0.78). The sampling and regulation periods are prefixed by the chip to 256 ms and 16 s, respectively. Then, the efficiency of the whole RF harvester (rectenna plus MPPT) was characterized by using the RF generator at the input of the rectenna and the SMU set at 3 V at the output of the MPPT (*V*_load_). The RF generator was set at different values of *P*_av_, from −20 dBm to −5 dBm in steps of 1 dBm, and for each value the SMU measured the output power *P*_load_. Then, from (23), *η*_T_ was estimated.

**Figure 6:**
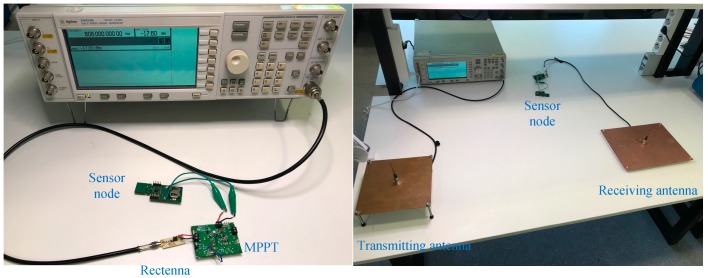
Picture of the setups for powering the sensor node using, for the RF harvester input, (**left**) the RF generator or (**right**) a monopole antenna.

For demonstration purposes, the RF harvester including the MPPT was also employed to power a sensor node intended to upgrade a mechanical gas meter to a smart device [38]. For these tests, the node was programmed to stay in a standby mode, consuming 1.4 µA. The input power (*P*_av_) was set to keep the voltage supply of the sensor node (*V*_load_) at 3 V, thus *P*_load_ = 4.2 µW. As for the RF harvester input, two configurations were used: (1) an RF generator and (2) a receiving monopole antenna. In the second case, another identical monopole antenna was connected to a nearby RF generator, jointly acting as a wireless energy transmitter. The antennas showed an insertion loss higher than 10 dB at 808 MHz. Figure 6 shows pictures of both setups.

## 5. Experimental Results and Discussion

**Figure 7:**
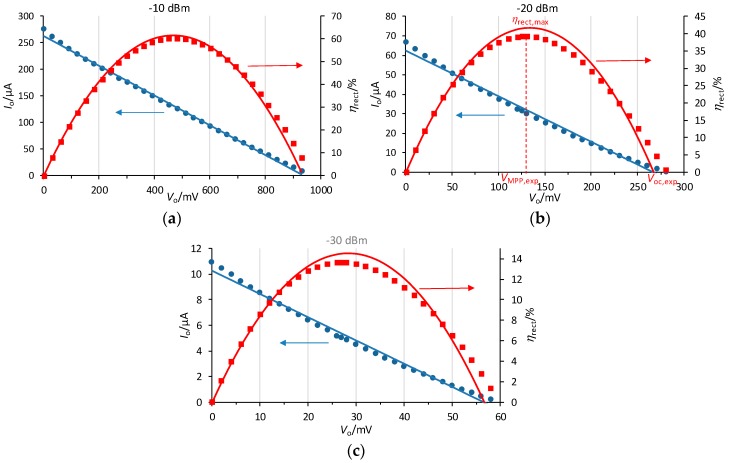
Measured values (dots) and least-squares fittings (continuous lines) of *I*_o_ and *η*_rect_ versus *V*_o_ for the rectenna at *P*_av_ equal to (**a**) −10 dBm, (**b**) −20 dBm, and (**c**) −30 dBm.

As for the proposed rectenna, Figure 7 shows the measured values (in dots) of *I*_o_ (blue circles) and *η*_rect_ (red squares) as a function of *V*_o_ at different values of *P*_av_. A least-squares fitting of (17) to the experimental data of *I*_o_ was performed (blue continuous line) to obtain the Thévenin parameters (*V*_oc_ and *R*_T_) at each power level, which are shown in Table 1. Calculated values of *V*_ap_, from (1), and of *V*_eqp_, from (15), are also included in Table 1. This fitting differs from that performed in [32], where the efficiency data (*η*_rect_) were used instead, which leads to slight differences in the Thévenin parameters. The new fitting procedure was considered more convenient as both *V*_oc_ and *R*_T_ can be more easily inferred from the fitting curve. As can be seen, the fitting curves match well the experimental data, and more at the highest power of −10 dBm, which confirms that the rectenna can be well approximated by a Thévenin equivalent circuit. Then, *V*_oc_ and *R*_T_ were used to obtain *η*_rect_ using (19), and the resulting curves are also represented in Figure 7 (red continuous line). The match with the experimental data is good, and again better at −10 dBm.

With *C*_m_ = 0.5 pF, *R*_a_ = 50 Ω, and *f*_o_ = 808 MHz, *Q* = 7.88 results from (3), and *G*_t_ = 3.97 from (2). Then, from (16) and assuming the value of *R*_p_ = 9.21 kΩ derived in Appendix A, *R*_T_ = 4.7 kΩ is obtained, which is within the range of values found in Table 1. The inferred values of *R*_T_ moderately change with *P*_av_ due to the relative low value of *Q*, which limits the accuracy of the rectenna model proposed in Section 2. However, a higher value of *Q*, which could be obtained using a lower value of *C*_m_ and appropriately readjusting *L*_m_, does not lead to the optimum gain *G*_t_ [33], thus decreasing the power efficiency. On the other hand, *V*_oc_ in Table 1 increases with increasing *P*_av_ and thus *V*_ap_, which agrees with (16). The values of *V*_oc_ can be estimated in advance, when necessary, from (16) by calculating *V*_eqp_ from (15), shown in Table 1, and inferring a value of *V*_γ_ from the manufacturer data or from simulations.

From the measured data of *η*_rect_ (red squares in Figure 7), Table 2 shows the achieved *η*_rect,max_ and its corresponding voltage (*V*_MPP,exp_), as well as the experimental open circuit voltage (*V*_oc,exp_) of the rectenna. In Figure 7, *η*_rect,max_, *V*_MPP,exp_, and *V*_oc,exp_ are also marked for *P*_av_ = −20 dBm. As can be seen, *η*_rect,max_ increases with increasing *P*_av_, ranging from 13.6% at −30 dBm to 60.3% at −10 dBm, which agrees with (21). The values of *η*_rect,max_ can be estimated in advance, when necessary, from (21) and inferring a value of *V*_γ_ from the manufacturer data or from simulations. One particular case is the upper limit, which would be achieved for *P*_av_→∞ (or *V*_γ_→0), in our case 74%. The resulting efficiencies (*η*_rect,max_) are among the highest published in the literature for similar designs [33]. On the other hand, *V*_oc_ from Table 1 nearly matches *V*_oc,exp_. Finally, *V*_MPP,exp_ equates or nearly matches 0.5 *V*_oc,exp_, the regulated voltage at the input of the MPPT. Thus, the proposed and implemented MPPT will be able to extract the maximum power (or nearly) from the rectenna.

**Figure 8:**
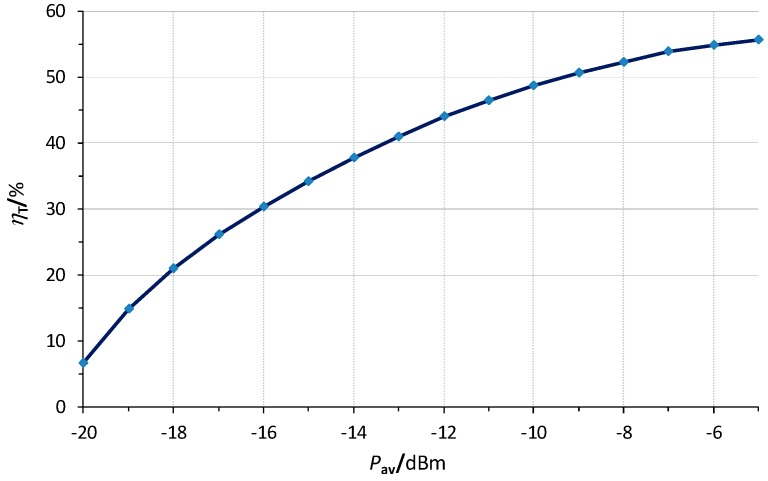
Overall efficiency (*η*_T_) of the RF harvester.

As for the whole RF harvester (rectenna plus the MPPT), Figure 8 shows the experimental values of *η*_T_ versus *P*_av_. At −20 dBm, *η*_rect,max_ = 39.3% (Table 2) but *η*_T_ = 6.5%, resulting, from (22), in *η*_MPPT_ = 16.5%. This low value of *η*_MPPT_ is due to both a low input voltage value (140 mV = 0.5 *V*_oc,exp_) and a low value of *P*_o_ (3.9 µW = *η*_rect,max_*P*_av_). Contrariwise, at −10 dBm, *η*_rect,max_ = 60.3% and *η*_T_ = 48.6%, resulting in *η*_MPPT_ = 80.6%, which agrees with the data from the BQ25504 chip’s datasheet. At higher values of *P*_av_ (−5 dBm), *η*_T_ reached a value of 55.6%. Compared to [6], where a similar chip for the MPPT was used, *η*_T_ is quite higher.

When powering the sensor node, the required value of *P*_av_ was −17.6 dBm. This value fits well with (23), considering the corresponding efficiency in Figure 8 (≈24%). This performance was also tested with the antennas at a distance of 0.5 and 1 m. The power output of the remote RF generator was tuned at appropriate values so as to operate the node, resulting in 8.0 and 13.2 dBm, respectively. These values accounted for the respective link budgets.

## 6. Conclusions

This work proposed a compact Thévenin model for a rectenna and its application for designing a high-efficiency RF harvester. The rectenna under study consists of an L-matching network and a half-wave rectifier. Explicit expressions for the Thévenin voltage and resistance were derived that offer insight into the operation of the rectenna. An expression was also provided for the power efficiency. The rectenna was implemented and characterized from −30 to −10 dBm at 808 MHz and the results mainly agreed with the derived model, with differences arising from the limited *Q* factor of the matching network. High efficiencies were obtained, in particular 60% at −10 dBm. Then, an ensuing MPPT was also added, where the behavior of the rectenna as an equivalent Thévenin circuit allowed the use of a simple FOCV technique. The whole RF harvester (rectenna plus MPPT) showed an overall efficiency near 50% at −10 dBm. Further tests were performed with a nearby transmitting antenna for powering a sensor node with a power consumption of 4.2 µW.

## Figures and Tables

**Table 1:** Inferred values of *V*_oc_ and *R*_T_ and calculated values of *V*_eqp_ at different values of *P*_av_.

*P*_av_ (dBm)	*V*_ap_ (mV)	*V*_oc_ (mV)	*R*_T_ (kΩ)	*V*_eqp_ (mV)
−10	200 mV	937	3.56	1183
−20	63.2 mV	268	4.29	374
−30	20.0 mV	56.6	5.51	118.3

**Table 2:** Experimental values of *η*_rect,max_, *V*_MPP,exp_, and *V*_oc,exp_ at different values of *P*_av_.

*P*_av_ (dBm)	*η*_rect,max_ (%)	*V*_MPP,exp_ (mV)	*V*_oc,exp_ (mV)
−10	60.3	480	960
−20	39.3	130	280
−30	13.6	27	60

**Figure A1:**
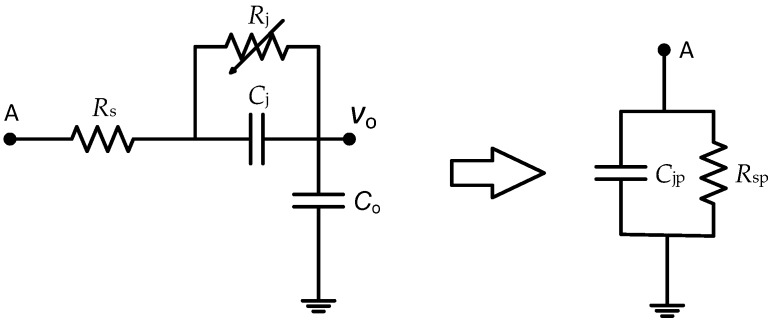
Equivalent linear circuit model of the HSMS-2850 diode together with (**left**) the output capacitor *C*_o_ and (**right**) its parallel equivalent circuit.

**Figure A2:**
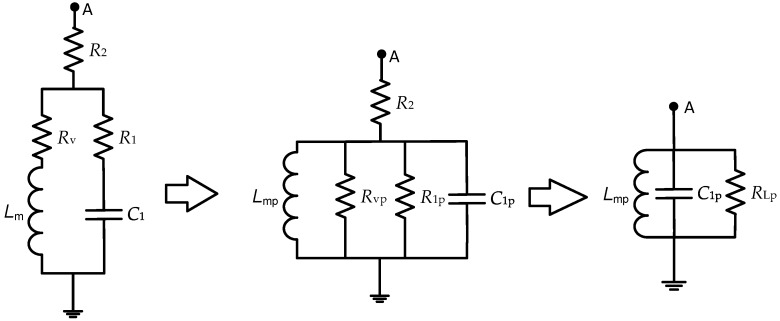
(**left**) Manufacturer model of the inductor and (**middle** and **right**) equivalent circuits.

**Figure A3:**
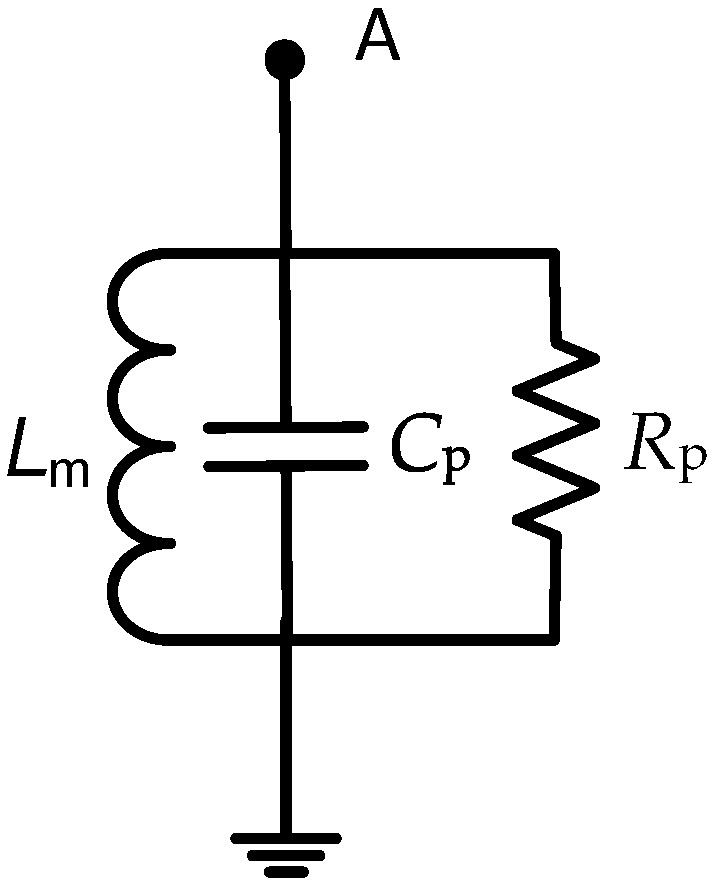
Parallel equivalent circuit of the inductor.

**Figure A4:**
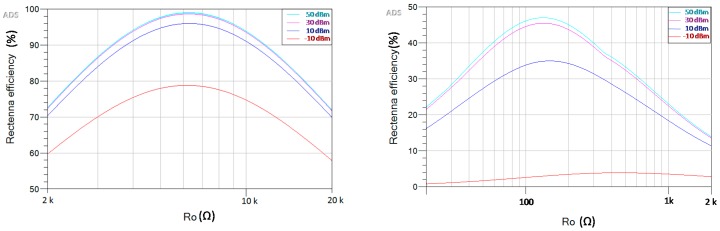
Rectenna efficiency for the circuit (**left**) with and (**right**) without the matching network.

## Appendix A. Parallel Circuit Model of the Inductor and Diode

The use of a matching network leads to sinusoidal voltage and current waveforms at *f*_o_, as mentioned in Section 2. Here, the equivalent circuit model at *f*_o_ is derived from the manufacturer models of the diode (https://docs.broadcom.com/docs/AV02-1377EN) and inductor (https://www.coilcraft.com/pdfs/spice_0603cs.pdf) used for the implemented rectenna and reported in Section 4. This model will be used in the left-hand circuit of Figure 3.

First, the left-hand circuit in Figure A1 shows the equivalent linear circuit model of the diode, connected between node A and the *V*_o_ node at Figure 2, together with the output capacitor *C*_o_, where *R*_s_ is the parasitic series resistance, *C*_j_ is the parasitic junction capacitance, and *R*_j_ is the junction resistance. *R*_j_ depends inversely on the bias current and makes only sense for modelling small current variations around a bias current. In our case, the diode current is pulsed and thus the inclusion of *R*_j_ is not appropriate. Instead, a constant voltage drop will be assumed in Section 2 for the analysis of the circuit of Figure 4.

Using the series-to-parallel equivalent circuit transformation for the left-hand circuit of Figure A1 without *R*_j_ and considering *C*_o_ >> *C*_j_, the right-hand circuit results, where:

(A1) $$R_{\mathrm{sp}}=R_{s}\frac{R_{s}^{2}C_{j}^{2}\omega_{o}^{2}+1}{R_{s}^{2}C_{j}^{2}\omega_{o}^{2}},$$

(A2) $$C_{\mathrm{jp}}=\frac{C_{j}}{R_{s}^{2}C_{j}^{2}\omega_{o}^{2}+1}.$$

From the diode datasheet, *R*_s_ = 25 Ω and *C*_jo_ = 0.18 pF (*C*_jo_ is *C*_j_ at zero bias and will be the assumed value for *C*_j_ hereafter). With *f*_o_ = 808 MHz, *R*_sp_ = 47.9 kΩ results from (A1) and *C*_jp_ ≈ *C*_j_ = 180 fF results from (A2).

As for the inductor, connected between node A and ground at Figure 2, the left-hand circuit of Figure A2 shows the manufacturer model, where $R_{v}=k\sqrt{f_{o}}$. Using the series-to-parallel circuit transformation, the circuit in the middle is obtained, where:

(A3) $$L_{\mathrm{mp}}=L_{m}\left[1+{\left(\frac{R_{v}}{\omega_{o}L_{m}}\right)}^{2}\right].$$

(A4) $$R_{\mathrm{vp}}=R_{v}\left[1+{\left(\frac{\omega_{o}L_{m}}{R_{v}}\right)}^{2}\right],$$

(A5) $$R_{1p}=R_{1}\frac{R_{1}^{2}C_{1}^{2}\omega_{o}^{2}+1}{R_{1}^{2}C_{1}^{2}\omega_{o}^{2}},$$

(A6) $$C_{1p}=\frac{C_{1}}{R_{1}^{2}C_{1}^{2}\omega_{o}^{2}+1}.$$

From the coil datasheet, *R*_1_ = 17 Ω, *R*_2_ = 30 mΩ, *C*_1_ = 49 fF, *L*_m_ = 27 nH, and *k* = 5.75 × 10^−5^. At 808 MHz, *R*_v_ = 1.63 Ω and from (A3) to (A6), *L*_mp_ ≈ *L*_m_ = 27 nH, *R*_vp_ = 11.5 kΩ, *R*_1p_ = 950 kΩ, and *C*_1p_ ≈ *C*_1_ = 49 fF. Then, neglecting *R*_2_, since it is very small, the right-hand circuit of Figure A2 is obtained, where $R_{\mathrm{Lp}}=R_{\mathrm{vp}}\|R_{1p}=11.4\;k\Omega$.

Joining the right-hand circuits in Figure A1 and Figure A2, and considering the parasitic capacitance from node A to ground arising from the layout (*C*_lay_), the circuit in Figure A3 is obtained, where:

(A7) $$R_{p}=R_{\mathrm{sp}}\|R_{\mathrm{Lp}}=9.21\;k\Omega ,$$

(A8) $$C_{p}=C_{\mathrm{jp}}+C_{1p}+C_{\mathrm{lay}}.$$

This equivalent circuit is used in the left-hand circuit of Figure 3.

## Appendix B. Simulations of the Rectenna Efficiency with and without an L-Matching Network

In order to highlight the benefits of using a matching network, we report simulation results of the rectenna efficiency with and without an L-matching network. For the simulations, the ADS software (Version 2017, Keysight, Santa Rosa, CA, USA) was used. The simulated circuit with the matching network was that of Figure 2 with a resistor *R*_o_ connected at its output, in parallel with *C*_o_. Ideal components were used for the matching network with the values reported in Section 4. The diode was modelled without parasitic elements (null series resistance and junction capacitance) but with a saturation current of 3 µA (that corresponding to the HSMS-2850 diode used for the implemented rectenna). For the RF source, a frequency of 808 MHz (that used for the experiments) was used with *P*_av_ ranging from −10 to 50 dBm in steps of 20 dB. For the circuit without the matching network, the right terminal of *R*_a_ was directly connected to the diode anode (node A). For both circuits, a harmonic balance analysis was performed with *P*_av_ and *R*_o_ as sweeping parameters.

Figure A4 shows the simulation results of the rectenna efficiency (*η*_rect_ = *P*_o_/*P*_av_) plotted against *R*_o_ for different values of *P*_av_. The left graph shows the results for the circuit with the L-matching network. As can be seen, *η*_rect_ steeply increases for increasing values of *P*_av_, achieving around 99% at *P*_av_ = 50 dBm. At −10 dBm, efficiency is higher than the corresponding results of Figure 7 because ideal components were used for the simulation. On the other hand, the right graph shows the results for the circuit without the L-matching network. As can be seen, *η*_rect_ steeply increases for increasing values of *P*_av_ but now reaches a maximum value around 46%, as predicted theoretically in [35], due to the additional losses at *R*_a_ originated by the current harmonics generated by the diode pulsed current. At −10 dBm, efficiency is lower than 4%. Therefore, the use of the matching network allows a notable increase in the rectenna efficiency, because it provides voltage gain and prevents any of the DC current and harmonics to flow through the antenna resistance.
